# Effect of Laterality in Microsurgery: Comparative Study of an Expert and a Novice

**DOI:** 10.3390/jcm13133894

**Published:** 2024-07-02

**Authors:** Célia Guttmann, Agata Durdzinska Timoteo, Sébastien Durand

**Affiliations:** Department of Hand Surgery, Lausanne University Hospital, Rue du Bugnon 46, CH-1011 Lausanne, Switzerland; celia.guttmann@unil.ch (C.G.); agata.timoteo@chuv.ch (A.D.T.)

**Keywords:** laterality, handedness, microsurgery, microsurgical anastomosis, skill learning

## Abstract

**Background:** Hand laterality has an impact on surgical gestures. In this study, we sought to measure the multi-parameter variability of the microsurgical gesture depending on the hand used and the differences between expert microsurgeons and novices. **Methods:** Ten experienced microsurgeons and twenty medical students with no prior microsurgical experience performed arterial anastomosis on a chicken wing artery using dominant and non-dominant hands. We measured time and force using a homemade force-sensing microsurgical needle holder, heart rate variability with a Polar H10 chest strap, anxiety with the STAI-Y questionnaire and anastomosis quality using the MARS 10 scale. **Results:** In the microsurgeons’ group, duration of anastomosis (*p* = 0.037), force applied to the needle holder (*p* = 0.047), anxiety (*p* = 0.05) and MARS10 (*p* = 0.291) were better with the dominant hand. For novices, there was no difference between the dominant and non-dominant hand pertaining to force, time and stress level. There were no differences between microsurgeons and novices pertaining to force and anxiety using the non-dominant hand. **Conclusions:** The study highlighted a marked laterality among microsurgical experts, a finding that may be explained by current learning methods. Surprisingly, no laterality is observed in students, suggesting that for a specific gesture completely different from everyday tasks, laterality is not predefined. Ambidexterity training in the residency curriculum seems relevant and may help microsurgeons improve performance and postoperative outcomes.

## 1. Introduction

Individuals’ preferential use of one hand is known as dominant hand (DH). Roughly 90% of the population exhibits right-hand dominance while a minority is left-hand dominant [1]. While the direction of handedness is categorical, the degree of consistency is continuous, ranging from comparable usage of both hands to unique usage of a single hand [2] and depends on the type of task. Ambidexterity is the ability to use the right and left hands equally well for all tasks, hence no hand dominance or preference [3]. The most recent systematic review on laterality suggests that the handedness phenomenon is a multifactorial process influenced by genetics, neural asymmetries, social pressure, and behavioural traits such as lateralized practice [4].

There is a lack of literature on handedness and its consequences in surgical training and practice [5]. Although usually regarded as a disadvantage for left-handed surgeons, the question of hand laterality may create problems for left- and right-handed surgeons [6]. For example, surgeon handedness appears to have an influence on the orientation of transpedicular screws in spinal surgery [7], on the acetabular component position during total hip replacement [8,9,10] and on the outcomes of total knee arthroplasties [11]. Adopting ambidexterity could also be a safe way of preserving the posture necessary for an injury-free surgical career [12]. Training in ambidexterity may help correct these technical surgical errors and is proposed by many authors [6,13,14,15].

Studies focusing on hand performance and preference after some days of motor task training with the non-dominant hand (NDH) showed that preference could be shifted to the non-dominant hand after practice, and that performance became symmetric between hands [16]. Training of the non-dominant hand can induce substantial and consistent improvement in the precision and quality of the NDH gesture [17] and can lead to improvement in the skills of the dominant side [18,19]. This interesting fact, already reported in the literature, is known as intermanual transfer of skill [20]. It has been demonstrated that attempting to switch handedness usually leads to some degree of ambidexterity, and not to a complete switch of behaviour [4].

Microsurgery involves very refined bimanual movements that predominantly employ the intrinsic muscles of the hands, requiring an operating microscope and a unique set of instruments to perform meticulous procedures [5]. In contrast to other surgical fields, 100% of the microsurgery instruments are ambidextrous [15]. This factor makes it an excellent discipline to appreciate bimanuality without the additional challenge of using instruments not adapted to one’s handedness. The main objective of this study is to measure the intra-individual variability and quality of the microsurgical gesture depending on the hand used and the differences between expert microsurgeons and novices.

## 2. Materials and Methods

### 2.1. Subjects

This study was approved by our local ethics committee: La Commission cantonale d’éthique de la recherche sur l’être humain (CER-VD, BASEC-ID 2020-00225). Ten experienced [21] or highly experienced microsurgeons (3 women and 7 men) with an average age of 45.1 years (SD 8.3 years) and twenty medical students (10 women and 10 men) with an average age of 26.2 years (SD 2.1 years) with no microsurgical experience were included. Exclusion criteria were individuals who suffered from pain or disability of the upper limbs or had medical conditions modifying their ability to perform precise manual tasks, those with a history of hand surgery and participants taking medications that can affect motor skills. One subject was left-handed in the group of microsurgeons and one in the students’ group.

### 2.2. Microsurgical Suture Protocol

Our model consisted of an end-to-end anastomosis performed on the brachial artery of a chicken wing [22] under a Zeiss OPMI 111 microscope at 12.5× magnification using non-resorbable Dafilon^®^ 10-0 DRm 4 black made of polyamide, non-resorbable. The needle is 3/8 of a circle (135°) with a 70 μ width. Microsurgical suture on a chicken wing artery using our connected microsurgical needle holder was performed with the dominant and non-dominant hands. For students, a macro-demonstration lesson was provided for end-to-end anastomosis according to Buncke [23]. All ten surgeons and ten students started the first suture with their dominant hand and then performed a second suture with their non-dominant hand. Ten other students performed the gesture first with their non-dominant hand and ended with their dominant hand.

### 2.3. Assessment Tools

#### 2.3.1. Force-Sensing Microsurgical Needle Holder

**Figure 1:**
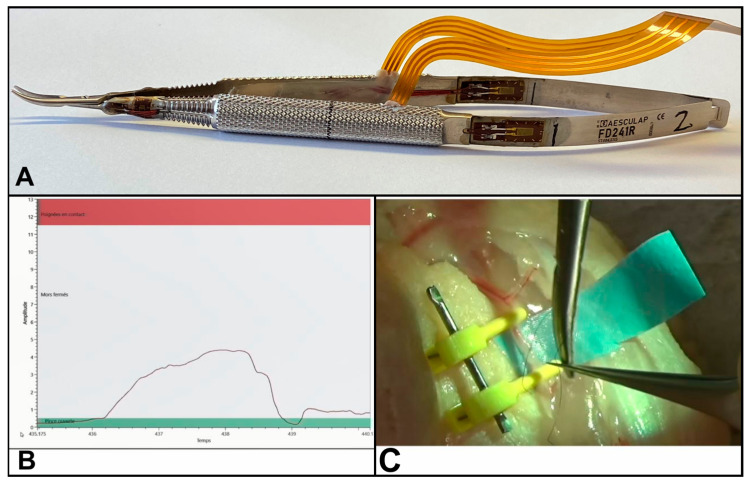
(**A**) Force-sensing microsurgical needle holder with eight strain gauges glued and soldered to a flex. Printed circuit. (**B**) Our software recorded in real time the force applied to the needle holder. (**C**) End-to-end anastomosis on chicken wing artery.

A microsurgical needle holder was equipped with sensors to measure time and force applied to the needle holder by hand during the microsurgical procedure. On a microsurgical needle holder FD 241 (Aesculap, Center Valley, PA, USA), eight strain gauges were glued and soldered to a flex-printed circuit. Four strain gauges were glued on the spring strips and four other strain gauges were glued between the screw and the grooves of each handle and placed on the outer side. Software was generated using LabVIEW NXG 2.1.3 to show in real time a graph of the force applied on the clamp and to record it with a 40 Hz frequency [24]. The interface was created on three levels: jaws open, jaws closed and the touching of the shafts of the body. These three levels were divided by two baselines: 0.8 N the minimal force needed to close the two jaws and 13 N the minimal force needed to touch the shafts of the body (Figure 1).

This tool was tested for validity and reliability. This tool was tightened by applying different forces from 0.1 to 13 N and the relative deviation between the force applied and the measured force was still under 3%. The phenomenon of creep resulting in a time-dependent change in its stress and deformation was evaluated. The microsurgical needle holder was tightened by applying 8.645 N force. After ten minutes, the measured force dropped to 8.408 N and a relative deviation of 2.74% was calculated.

#### 2.3.2. State-Trait Anxiety Inventory (STAI) Questionnaire

Anxiety was evaluated using the State-Trait Anxiety Inventory (STAI) questionnaire. The first questionnaire (STAI Form Y-1) was used and measures how participants feel “at this moment”, which allows evaluation of the nervousness and anxiety during the session (for example “I feel calm, I feel nervous”) [25].

#### 2.3.3. Heart Rate Variability (HRV) Measurement Using Polar H10

HRV is the physiological phenomenon describing the variation in time between consecutive heartbeats (RR interval) in milliseconds [26]. As the sympathetic nervous system begins to supplant the parasympathetic nervous system, there occurs less interplay between these two autonomic nervous system divisions, which manifests as a near-instantaneous decrease in RR interval variation (lower HRV) suggesting a high-level of sympathetic nervous system activation referred to as stress [27]. In addition to the ease of measurability, reproducibility and sensitivity sufficient to observe the nearly instantaneous changes in the autonomic nervous system balance [28], HRV makes an excellent marker to compare stress rates [29]. Polar H10 (Polar Electro Oy, Kempele, Finland) chest strap heart-rate sensors have been validated and used to assess RR intervals during rest and physical exercise [30]. Compared to ECG, Polar H10 is accepted for the measurement of HRV [31].

#### 2.3.4. Microsurgical Anastomosis Rating Scale

The Microsurgical Anastomosis Rating Scale (MARS10) score is a validated tool for assessing microsurgical end-to-end arterial anastomoses on non-living models [32]. It consists of 5 items (anastomosis closure, suture spacing, bite-size, knot tying and cut end length), graded on a 3-point scale (0–2 points). A blinded evaluation was carried out for each expert anastomosis by two experts in microsurgery.

### 2.4. Statistical Analysis

IBM SPSS software v29.0.2.0 was used for data and statistical analysis. Statistical analysis was performed using the Wilcoxon signed-rank test and the Spearman correlation test. A *p*-value < 0.05 was considered statistically significant.

Using results of suture time and maximal force parameters from our previous publication [33] and with a statistical power of 90% and a significance level α = 0.05, 10 microsurgeons and 10 students were sufficient to observe a statistical difference. A total of 20 students were included to eliminate bias related to intermanual transfer of skill (10 students started the first suture with their dominant hand while 10 other students performed the gesture with their non-dominant hand first).

## 3. Results

### 3.1. Time

**Figure 2:**
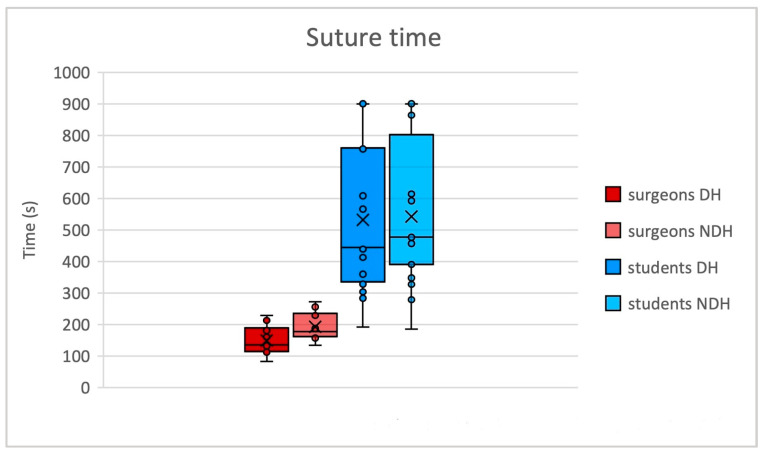
Suture time (seconds) differences between surgeons and students. DH: dominant hand; NDH: non-dominant hand.

In the surgeon’s group, we observed (Figure 2) a significant difference (*p* = 0.037) in the duration of suture between DH (mean 148.9 s, SD 46.3 s) and NDH (mean 192.7 s, SD 45.4 s). For students, the difference between DH and NDH was not statistically significant (*p* = 0.872). Time of suture was significantly shorter for surgeons compared to students (*p* < 0.01).

### 3.2. Force

**Figure 3:**
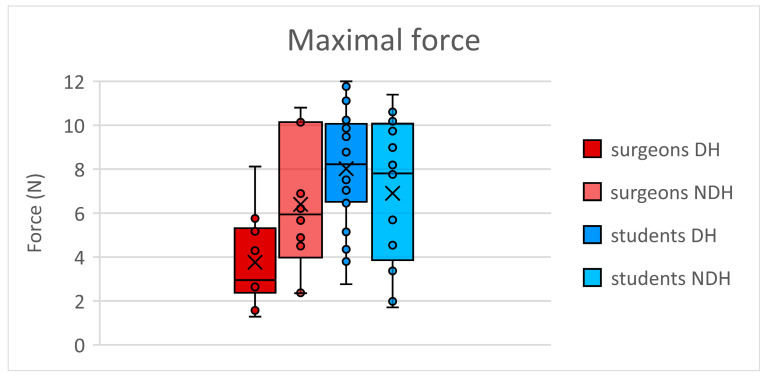
Maximal force (Newtons) applied on the needle holder. DH: dominant hand; NDH: non-dominant hand.

In the surgeon’s group, the maximal force applied to the needle holder (Figure 3) was significantly greater (*p* = 0.047) with the NDH (mean 6.4 N, SD 3.1 N) compared to the DH (mean 3.7 N, SD 2.1 N). For students, we observed no significant differences (*p* = 0.117) in the force applied on the needle holder between the DH (mean 8.0 N, SD 2.6 N) and the NDH (mean 6.9, SD 3.1 N).

### 3.3. Anxiety

**Figure 4:**
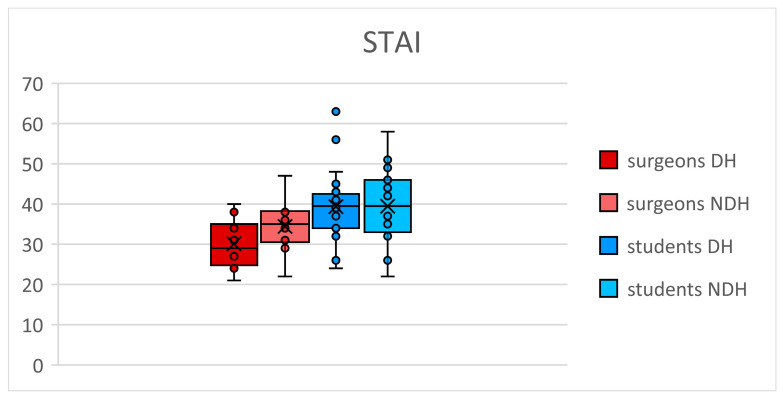
State-Trait Anxiety Inventory (STAI) questionnaire. DH: dominant hand; NDH: non-dominant hand.

For microsurgeons, we observed a significant increase (*p* = 0.05) in STAI (Figure 4) with the NDH (mean 34.4, SD 6.7) compared to the DH (mean 30.1, SD 6.3). For students, STAI was not statistically different (*p* = 0.87) between the DH (mean 39.3, SD 9.1) and the NDH (mean 39.2, SD 9.4).

### 3.4. Heart Rate Variability

**Figure 5:**
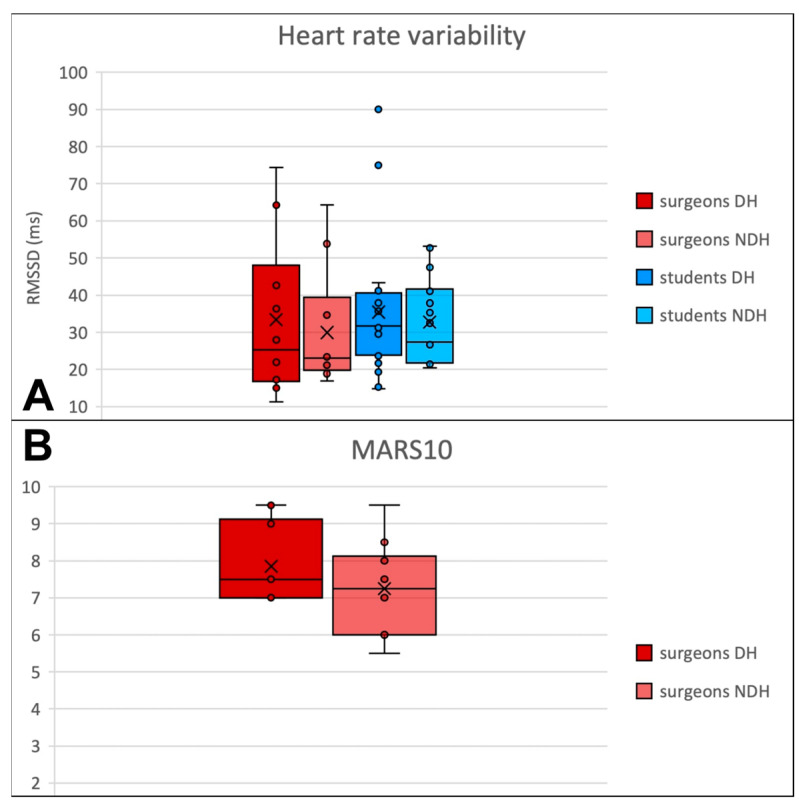
(**A**) The root mean square of successive differences between normal heartbeats (RMSSD). (**B**) Mars 10 score in the surgeon’s group. DH: dominant hand; NDH: non-dominant hand.

For microsurgeons, we observed no significant difference (*p* = 0.678) in HRV (Figure 5a) between the DH (mean 33.4 ms, SD 21.3 ms) and the NDH (mean 29.9 ms, 16.2 ms).

For students, we observed no significant difference (*p* = 0.881) in the HRV between the DH (mean 35.4 ms, SD 18.4 ms) and the NDH (mean 32.7 ms, SD 11.2 ms).

### 3.5. Suture Quality

Mars 10 score was better (*p* = 0.291) with the DH (mean 7.8, SD 1.1) compared to the NDH (mean 7.2, SD 1.2) among microsurgeons (Figure 5b). For ethical reasons, complete suture was not performed by students.

### 3.6. Microsurgeons vs. Students

Differences between microsurgeons and students are summarized in Table 1.

We observed no differences in the NDH pertaining to maximal force and STAI (*p* = 0.878 and *p* = 0.720, respectively), but a significant difference in the DH between maximal force and STAI (*p* = 0.005 and *p* = 0.05, respectively). Suture time was significantly shorter for microsurgeons (*p* < 0.01), regardless of the hand used.

### 3.7. Students vs. Students

For the ten students who started the exercise with their DH, the time needed for suture and the force applied to the needle holder tended to be greater with the DH. With their NDH, the same ten students had significantly lower heart rate variability (*p* = 0.037) and anxiety scores (*p* = 0.028). For the ten other students who started the exercises with their NDH, the trends were reversed and no results were statistically different.

## 4. Discussion

This study highlighted a marked laterality among microsurgical experts; Time, force, anxiety and MARS10 scores were worse with the NDH compared to the DH. Laterality is rarely taken into account during learning, teaching or training in microsurgery [34] and can explain the better results in the DH in the microsurgeons’ group. Surprisingly, no laterality was observed in the students’ group. There is no significant difference between the DH and the NDH based on time, force, anxiety or HRV. This suggests that for a difficult bimanual gesture, completely different from everyday tasks, laterality is not predefined. This hypothesis opens the door to facilitated ambidexterity acquisition in microsurgery through the development of the skills and training of both hands.

Average force appeared lower among students due to the extended duration of their performance, as the forceps were frequently in an open position, registering a value of 0 N. This is the reason why the maximal force parameter was more relevant [33]. The maximal force applied to the needle holder seems to be correlated with a higher anxiety score. This indicates that a surgeon applying less force achieves better anastomosis. Relaxation is the physical and mental change resulting from a decrease in muscle and nervous tension and a generalized diminution of neurophysiological excitation [35]. Surgical performance can be enhanced by relaxation and lowering the stress level [36,37]. Relaxation can also reduce tremors, which is a known factor that negatively influences microsurgical efficiency [38].

The subject of handedness in surgery has received surprisingly scant attention in the surgical literature [39]. The consequence of laterality is especially important for many surgical procedures that are commonly set up for a right-handed approach. In a survey of surgeons on handedness in endoscopic surgery, 50% of left-handed surgeons believed that the laparoscopic approach should be modified for the left-handed individual [40]. Currently, no recommendations exist to improve training and operative approaches for left-handed surgeons.

It has been postulated that both our hands are not just better and worse versions of the same tool, but each has its own field of excellence. The DH has better precision in trajectory and speed precision, direction planification, muscle activation optimisation and feed-forward for trajectory control. The NDH, on the other hand, excels at final position precision, object stabilisation and feedback for position control [41], which explains the results observed in students with no microsurgery experience. Microsurgical gesture demands precise stabilization and constant position feedback. Understanding the nuanced contributions of each hand in microsurgical procedures can be pivotal in refining training methodologies and improving overall proficiency in this specialized field. The different specialisations of each hand are also the underlying basis of the intermanual transfer of skills. This phenomenon occurs when training of one hand also enhances the performance of the other hand, but each hand improves only particular features of the movement that are attributed to it. This means that even if the surgeon continues to use his DH to suture, his performance can be enhanced by training his NDH [41]. Essentially, both hands must work symbiotically and therefore the training of both the NDH and the DH is crucial.

All surgeons and microsurgeons are fully aware of the importance of ambidexterity. If controversy exists concerning the results of simulated NDH microsurgery [42], including the NDH in microsurgery training could help to develop ambidexterity for this task, which might be very important in some clinical settings [13]. It has been evocated that many microsurgical procedures require mixed handedness for a faster, smoother, and better postoperative outcome [43]. Incorporation of deliberate repetitive practice with the NDH into the postgrade formation and fundamentals of surgical simulations (open and laparoscopic/thoracoscopic) are potential approaches [6].

Even if expert surgeons exhibit laterality, a considerable amount of research concludes that experts have a higher rate of use of the NDH and bimanual gestures [44] and need a certain degree of ambidexterity. In a recent and very interesting publication, it has been demonstrated that robotic procedures require less ambidexterity than laparoscopic techniques, while other surgical techniques such as microsurgery require more ambidexterity [45]. It is possible that ambidexterity, with the evolution of surgical techniques, will only be useful for certain surgical procedures and its necessity may decrease with time.

Some limitations of this study must be mentioned. The mean lengths of time for the HRV assessment were 148.7 s and 192.7 s for microsurgeons and 531.5 s and 541.9 s for students. HRV can be conducted over short times, but those readings are not as accurate or precise. For ethical reasons, complete suture was not performed by students. Many students were exhausted after a few minutes of using the microscope and it did not seem reasonable to continue the test for more than 15 min. Comparison of the quality of suture between microsurgeons and students using Mars 10 was not possible.

## 5. Conclusions

This study highlighted a marked laterality among microsurgical experts, a finding that may be explained by current learning methods. Surprisingly, no laterality was observed in students, suggesting that for a specific gesture completely different from everyday tasks, laterality is not predefined. Ambidexterity training in postgrade formation and surgical simulation seems relevant and may help microsurgeons to improve performance and postoperative outcomes.

## Figures and Tables

**Table 1:** Results of the analyses.

Participants	Males	Females	Age (y)	Mean Time (s)		Mean Force (N)		Mean Anxiety (STAI)		Mean HRV R-R int (ms)		Mean Suture Quality (Mars 10)	
				DH	NDH	DH	NDH	DH	NDH	DH	NDH	DH	NDH
Microsurgeon	**7**	**3**	45.1	148.9	192.7	3.7	6.4	30.1	34.4	33.4	29.9	7.8	7.2
Student	10	10	26.2	531.5	541.9	8.0	6.9	39.3	39.2	35.4	32.7	-	-

DH: dominant hand; NDH: non-dominant hand; N: Newtons; s: seconds; ms: milliseconds.

## Data Availability

All data generated or analysed in this study are included in this article. Further enquiries can be directed to the corresponding author.
